# Supplementary material for: Altered microRNA Transcriptome in Cultured Human Liver Cells upon Infection with Ebola Virus
Source: Int J Mol Sci. 2021 Apr 6;22(7):3792. doi: 10.3390/ijms22073792 (PMC8038836; doi:10.3390/ijms22073792)
Supplement: Supplementary file 1 [file ijms-22-03792-s001.zip › Supplementary_File/C_ GO_Analysis_Results/16-30nt_go_Reston-24h-Huh7_vs_Control-24h-Huh7_down.mature_mirna_targets/BP_result(Human).html]

| GO.ID | Term | Ontology | Count | Pop.Hits | List.Total | Pop.Total | Fold.Enrichment | Pvalue | FDR | Enrichment.Score | Gene.Ratio | GENES |
| --- | --- | --- | --- | --- | --- | --- | --- | --- | --- | --- | --- | --- |
| GO:0001961 | positive regulation of cytokine-mediated signaling pathway | Biological process | 4 | 46 | 175 | 17653 | 8.77167701863354 | 0.00110057204452075 | 1 | 2.95838152283921 | 0.0228571428571429 | MAVS//CSF1//TRIM44//TSLP// |
| GO:0060760 | positive regulation of response to cytokine stimulus | Biological process | 4 | 50 | 175 | 17653 | 8.06994285714286 | 0.00150621442819223 | 1 | 2.82211319656056 | 0.0228571428571429 | TRIM44//TSLP//MAVS//CSF1// |
| GO:0010977 | negative regulation of neuron projection development | Biological process | 6 | 138 | 175 | 17653 | 4.38583850931677 | 0.00254623823641537 | 1 | 2.59410096442151 | 0.0342857142857143 | SEMA3F//RHOA//THY1//EFNA1//STMN2//RIT2// |
| GO:0035725 | sodium ion transmembrane transport | Biological process | 6 | 147 | 175 | 17653 | 4.11731778425656 | 0.00348270579024495 | 1 | 2.45808321203903 | 0.0342857142857143 | CHP1//ATP1B2//FXYD2//SLC9B1//SLC24A3//SCN4A// |
| GO:0035637 | multicellular organismal signaling | Biological process | 7 | 199 | 175 | 17653 | 3.54834170854271 | 0.00374560084431272 | 1 | 2.42647850575117 | 0.04 | CNTNAP1//SCN4A//ATP1B2//CTNNA3//ASPH//FXYD2//STIM1// |
| GO:0045792 | negative regulation of cell size | Biological process | 2 | 10 | 175 | 17653 | 20.1748571428571 | 0.00417324291860892 | 1 | 2.3795263348807 | 0.0114285714285714 | RHOA//DEPTOR// |
| GO:0035330 | regulation of hippo signaling | Biological process | 2 | 11 | 175 | 17653 | 18.3407792207792 | 0.00506751128275316 | 1 | 2.29520527568242 | 0.0114285714285714 | WWC3//NF2// |
| GO:0061001 | regulation of dendritic spine morphogenesis | Biological process | 3 | 37 | 175 | 17653 | 8.17899613899614 | 0.0058099383746877 | 1 | 2.23582847409418 | 0.0171428571428571 | EFNA1//BAIAP2//LRP8// |
| GO:0017156 | calcium ion regulated exocytosis | Biological process | 5 | 116 | 175 | 17653 | 4.34802955665025 | 0.00598717258897565 | 1 | 2.22277822250248 | 0.0285714285714286 | SYT2//VAMP2//DNAJC5//DOC2A//CHP1// |
| GO:0021559 | trigeminal nerve development | Biological process | 2 | 12 | 175 | 17653 | 16.812380952381 | 0.00604156022610195 | 1 | 2.21885089083505 | 0.0114285714285714 | SEMA3F//POU4F1// |
| GO:0035329 | hippo signaling | Biological process | 3 | 38 | 175 | 17653 | 7.96375939849624 | 0.00626255186477602 | 1 | 2.20324866439931 | 0.0171428571428571 | NF2//WWC3//TEAD3// |
| GO:0050767 | regulation of neurogenesis | Biological process | 15 | 732 | 175 | 17653 | 2.06709601873536 | 0.00628869312509279 | 1 | 2.20143959740542 | 0.0857142857142857 | STMN2//RIT2//NF2//RHOA//ZC4H2//SEMA3F//EFNA1//THY1//CAMSAP2//BAIAP2//LRP8//SYT2//CSF1//ZNF609//POU4F1// |
| GO:0031345 | negative regulation of cell projection organization | Biological process | 6 | 167 | 175 | 17653 | 3.62422583404619 | 0.00645285190255688 | 1 | 2.19024830213918 | 0.0342857142857143 | STMN2//RIT2//SEMA3F//RHOA//THY1//EFNA1// |
| GO:1903779 | regulation of cardiac conduction | Biological process | 4 | 75 | 175 | 17653 | 5.37996190476191 | 0.00656605369154705 | 1 | 2.18269557030493 | 0.0228571428571429 | ASPH//ATP1B2//FXYD2//STIM1// |
| GO:0014003 | oligodendrocyte development | Biological process | 3 | 39 | 175 | 17653 | 7.75956043956044 | 0.00673564551931478 | 1 | 2.17162077677213 | 0.0171428571428571 | CNTNAP1//FA2H//HDAC11// |
| GO:0010248 | establishment or maintenance of transmembrane electrochemical gradient | Biological process | 2 | 13 | 175 | 17653 | 15.5191208791209 | 0.00709373848495658 | 1 | 2.14912482610284 | 0.0114285714285714 | ATP1B2//FXYD2// |
| GO:0007019 | microtubule depolymerization | Biological process | 3 | 40 | 175 | 17653 | 7.56557142857143 | 0.00722944234766098 | 1 | 2.14089520127726 | 0.0171428571428571 | STMN2//CAMSAP2//KIF18B// |
| GO:0097286 | iron ion import | Biological process | 2 | 14 | 175 | 17653 | 14.410612244898 | 0.00822241936844289 | 1 | 2.08500037664884 | 0.0114285714285714 | STEAP2//ARHGAP1// |
| GO:1901224 | positive regulation of NIK/NF-kappaB signaling | Biological process | 3 | 43 | 175 | 17653 | 7.03774086378738 | 0.0088370908870234 | 1 | 2.05369067838553 | 0.0171428571428571 | MAS1//AGO1//TRIM44// |
| GO:0006000 | fructose metabolic process | Biological process | 2 | 15 | 175 | 17653 | 13.4499047619048 | 0.00942600043815993 | 1 | 2.02567254438856 | 0.0114285714285714 | PFKFB3//FBP2// |
| GO:0036376 | sodium ion export across plasma membrane | Biological process | 2 | 15 | 175 | 17653 | 13.4499047619048 | 0.00942600043815993 | 1 | 2.02567254438856 | 0.0114285714285714 | ATP1B2//FXYD2// |
| GO:0034755 | iron ion transmembrane transport | Biological process | 2 | 16 | 175 | 17653 | 12.6092857142857 | 0.0107029031917079 | 1 | 1.97049840277086 | 0.0114285714285714 | STEAP2//ARHGAP1// |
| GO:0050884 | neuromuscular process controlling posture | Biological process | 2 | 16 | 175 | 17653 | 12.6092857142857 | 0.0107029031917079 | 1 | 1.97049840277086 | 0.0114285714285714 | POU4F1//CNTNAP1// |
| GO:0048013 | ephrin receptor signaling pathway | Biological process | 4 | 87 | 175 | 17653 | 4.63789819376026 | 0.0109764306652739 | 1 | 1.95953886156054 | 0.0228571428571429 | ARPC3//VAV3//EFNA1//RHOA// |
| GO:0016079 | synaptic vesicle exocytosis | Biological process | 4 | 88 | 175 | 17653 | 4.5851948051948 | 0.0114122393825652 | 1 | 1.94262912717998 | 0.0228571428571429 | SYT2//DOC2A//VAMP2//DNAJC5// |
| GO:0050773 | regulation of dendrite development | Biological process | 5 | 136 | 175 | 17653 | 3.70861344537815 | 0.011482587676861 | 1 | 1.93996022978275 | 0.0285714285714286 | EFNA1//BAIAP2//LRP8//CAMSAP2//RHOA// |
| GO:0010771 | negative regulation of cell morphogenesis involved in differentiation | Biological process | 4 | 89 | 175 | 17653 | 4.53367576243981 | 0.0118590371260146 | 1 | 1.9259505713288 | 0.0228571428571429 | SEMA3F//RHOA//THY1//EFNA1// |
| GO:0043401 | steroid hormone mediated signaling pathway | Biological process | 6 | 191 | 175 | 17653 | 3.16882572924458 | 0.0120504659067865 | 1 | 1.91899616165054 | 0.0342857142857143 | NR6A1//RHOA//NCOA4//UFM1//ESRRG//VDR// |
| GO:0022010 | central nervous system myelination | Biological process | 2 | 17 | 175 | 17653 | 11.8675630252101 | 0.0120515727500508 | 1 | 1.91895627324697 | 0.0114285714285714 | FA2H//CNTNAP1// |
| GO:0032291 | axon ensheathment in central nervous system | Biological process | 2 | 17 | 175 | 17653 | 11.8675630252101 | 0.0120515727500508 | 1 | 1.91895627324697 | 0.0114285714285714 | CNTNAP1//FA2H// |
| GO:0031647 | regulation of protein stability | Biological process | 7 | 253 | 175 | 17653 | 2.79098814229249 | 0.0132419885513991 | 1 | 1.87804679193169 | 0.04 | PIM2//CHP1//EFNA1//ATP1B2//TRIM44//ASPH//NF2// |
| GO:0098659 | inorganic cation import across plasma membrane | Biological process | 3 | 50 | 175 | 17653 | 6.05245714285714 | 0.0133463229471271 | 1 | 1.87463837053438 | 0.0171428571428571 | STEAP2//ARHGAP1//FXYD2// |
| GO:0099587 | inorganic ion import across plasma membrane | Biological process | 3 | 50 | 175 | 17653 | 6.05245714285714 | 0.0133463229471271 | 1 | 1.87463837053438 | 0.0171428571428571 | STEAP2//ARHGAP1//FXYD2// |
| GO:0002363 | alpha-beta T cell lineage commitment | Biological process | 2 | 18 | 175 | 17653 | 11.208253968254 | 0.0134704775486688 | 1 | 1.87061700761217 | 0.0114285714285714 | SLAMF6//RHOA// |
| GO:0045655 | regulation of monocyte differentiation | Biological process | 2 | 18 | 175 | 17653 | 11.208253968254 | 0.0134704775486688 | 1 | 1.87061700761217 | 0.0114285714285714 | ZBTB46//CSF1// |
| GO:0061003 | positive regulation of dendritic spine morphogenesis | Biological process | 2 | 18 | 175 | 17653 | 11.208253968254 | 0.0134704775486688 | 1 | 1.87061700761217 | 0.0114285714285714 | BAIAP2//LRP8// |
| GO:1902307 | positive regulation of sodium ion transmembrane transport | Biological process | 2 | 18 | 175 | 17653 | 11.208253968254 | 0.0134704775486688 | 1 | 1.87061700761217 | 0.0114285714285714 | CHP1//ATP1B2// |
| GO:0061337 | cardiac conduction | Biological process | 5 | 143 | 175 | 17653 | 3.52707292707293 | 0.0140224393021373 | 1 | 1.85317643121175 | 0.0285714285714286 | ATP1B2//CTNNA3//ASPH//FXYD2//STIM1// |
| GO:0060997 | dendritic spine morphogenesis | Biological process | 3 | 51 | 175 | 17653 | 5.93378151260504 | 0.0140790990775933 | 1 | 1.85142513483511 | 0.0171428571428571 | EFNA1//BAIAP2//LRP8// |
| GO:0010975 | regulation of neuron projection development | Biological process | 10 | 450 | 175 | 17653 | 2.24165079365079 | 0.0144545870953594 | 1 | 1.83999430971924 | 0.0571428571428571 | STMN2//RIT2//SEMA3F//EFNA1//RHOA//THY1//CAMSAP2//BAIAP2//LRP8//SYT2// |
| GO:0045665 | negative regulation of neuron differentiation | Biological process | 6 | 203 | 175 | 17653 | 2.98150598170303 | 0.0158544866183732 | 1 | 1.79984781623023 | 0.0342857142857143 | STMN2//RIT2//SEMA3F//RHOA//THY1//EFNA1// |
| GO:0045649 | regulation of macrophage differentiation | Biological process | 2 | 20 | 175 | 17653 | 10.0874285714286 | 0.0165129813543299 | 1 | 1.78217450948446 | 0.0114285714285714 | ZBTB46//CSF1// |
| GO:0051960 | regulation of nervous system development | Biological process | 15 | 820 | 175 | 17653 | 1.84526132404181 | 0.0165427874763793 | 1 | 1.78139130955906 | 0.0857142857142857 | STMN2//RIT2//NF2//RHOA//ZC4H2//SEMA3F//POU4F1//EFNA1//THY1//CAMSAP2//BAIAP2//LRP8//SYT2//CSF1//ZNF609// |
| GO:0032722 | positive regulation of chemokine production | Biological process | 3 | 55 | 175 | 17653 | 5.50223376623377 | 0.0172354950017709 | 1 | 1.76357623918086 | 0.0171428571428571 | MAVS//TSLP//FFAR3// |
| GO:0016192 | vesicle-mediated transport | Biological process | 30 | 2050 | 175 | 17653 | 1.47620905923345 | 0.0185267257885537 | 1 | 1.73220132636962 | 0.171428571428571 | VAV3//SYT2//VAMP2//DOC2A//CDK16//DNAJC5//KDELR1//ZW10//ARF3//SCAMP2//STEAP2//LRP8//CLEC9A//VIPAS39//ARHGAP1//CHP1//RIT2//TBC1D9B//ARPC3//BAIAP2//RAB9B//SIRPB1//LRG1//CR1//RHOA//SIRPG//STARD3//SMPD3//ANKRD50//MAPK8IP1// |
| GO:1900180 | regulation of protein localization to nucleus | Biological process | 4 | 102 | 175 | 17653 | 3.95585434173669 | 0.0187075332955926 | 1 | 1.72798347314867 | 0.0228571428571429 | MAVS//CHP1//TRIM29//NF2// |
| GO:0032271 | regulation of protein polymerization | Biological process | 6 | 211 | 175 | 17653 | 2.86846310088016 | 0.0188094719567347 | 1 | 1.72562339634198 | 0.0342857142857143 | BAIAP2//RHOA//CAMSAP2//STMN2//TPPP3//ARPC3// |
| GO:0051261 | protein depolymerization | Biological process | 4 | 103 | 175 | 17653 | 3.9174479889043 | 0.0193171052346827 | 1 | 1.71405795425574 | 0.0228571428571429 | STMN2//KIF18B//CAMSAP2//ASPH// |
| GO:0048522 | positive regulation of cellular process | Biological process | 64 | 5142 | 175 | 17653 | 1.25553370006112 | 0.0196445571092071 | 1 | 1.70675775800492 | 0.365714285714286 | PEA15//EFNA1//MAVS//CDK2AP1//LRG1//TRIM44//TSLP//THY1//MAS1//MAP3K9//CSF1//SIRPG//VAV3//ARHGAP1//CNTNAP1//SPDEF//PIM2//PRODH//STMN2//RIT2//PIK3AP1//RHOA//ESRRG//ANKRD49//NCOA4//SEMA3F//BAIAP2//HLA-DPA1//NDFIP2//TPPP3//ARPC3//FGD1//ADAMTSL4//POU4F1//EEF1E1//MAPK8IP1//NF2//VDR//ZC4H2//PFKFB3//ASPH//PSMC6//PRDM4//ZNF609//GABPA//NR6A1//AGO1//PROX2//HLF//MAFG//TEAD3//ZNF24//CLEC9A//LRP8//SIRPB1//CHP1//VAMP2//CCL16//CR1//ATP1B2//SMPD3//SYT2//ZBTB46//FFAR3// |
| GO:0018230 | peptidyl-L-cysteine S-palmitoylation | Biological process | 2 | 22 | 175 | 17653 | 9.17038961038961 | 0.0198186168812635 | 1 | 1.70292665771124 | 0.0114285714285714 | ZDHHC19//ZDHHC20// |
| GO:0018231 | peptidyl-S-diacylglycerol-L-cysteine biosynthetic process from peptidyl-cysteine | Biological process | 2 | 22 | 175 | 17653 | 9.17038961038961 | 0.0198186168812635 | 1 | 1.70292665771124 | 0.0114285714285714 | ZDHHC19//ZDHHC20// |
| GO:0099175 | regulation of postsynapse organization | Biological process | 3 | 58 | 175 | 17653 | 5.2176354679803 | 0.019840995553798 | 1 | 1.70243654021241 | 0.0171428571428571 | EFNA1//BAIAP2//LRP8// |
| GO:0031109 | microtubule polymerization or depolymerization | Biological process | 4 | 104 | 175 | 17653 | 3.87978021978022 | 0.0199388467304851 | 1 | 1.70029996503495 | 0.0228571428571429 | STMN2//KIF18B//CAMSAP2//TPPP3// |
| GO:0043244 | regulation of protein complex disassembly | Biological process | 4 | 105 | 175 | 17653 | 3.84282993197279 | 0.0205728175055322 | 1 | 1.6867062263747 | 0.0228571428571429 | ETF1//STMN2//CAMSAP2//ASPH// |
| GO:1902305 | regulation of sodium ion transmembrane transport | Biological process | 3 | 59 | 175 | 17653 | 5.129200968523 | 0.0207550755889218 | 1 | 1.68287568060433 | 0.0171428571428571 | CHP1//ATP1B2//FXYD2// |
| GO:0050821 | protein stabilization | Biological process | 5 | 159 | 175 | 17653 | 3.17214734950584 | 0.0211840825811842 | 1 | 1.67399033924178 | 0.0285714285714286 | PIM2//CHP1//EFNA1//ATP1B2//TRIM44// |
| GO:0001952 | regulation of cell-matrix adhesion | Biological process | 4 | 106 | 175 | 17653 | 3.80657681940701 | 0.0212190749230521 | 1 | 1.6732735537321 | 0.0228571428571429 | NF2//CSF1//RHOA//THY1// |
| GO:0007026 | negative regulation of microtubule depolymerization | Biological process | 2 | 23 | 175 | 17653 | 8.77167701863354 | 0.021566519270653 | 1 | 1.66621994221916 | 0.0114285714285714 | STMN2//CAMSAP2// |
| GO:0018126 | protein hydroxylation | Biological process | 2 | 23 | 175 | 17653 | 8.77167701863354 | 0.021566519270653 | 1 | 1.66621994221916 | 0.0114285714285714 | ASPH//VIPAS39// |
| GO:0140115 | export across plasma membrane | Biological process | 2 | 23 | 175 | 17653 | 8.77167701863354 | 0.021566519270653 | 1 | 1.66621994221916 | 0.0114285714285714 | ATP1B2//FXYD2// |
| GO:1901222 | regulation of NIK/NF-kappaB signaling | Biological process | 3 | 60 | 175 | 17653 | 5.04371428571429 | 0.0216919789590337 | 1 | 1.66370082549695 | 0.0171428571428571 | MAS1//AGO1//TRIM44// |
| GO:0002682 | regulation of immune system process | Biological process | 24 | 1579 | 175 | 17653 | 1.53323803492265 | 0.0231665128622358 | 1 | 1.63513933343764 | 0.137142857142857 | MAVS//PSMC6//TRIM44//CR1//THY1//FFAR3//CSF1//HLA-DPA1//ZBTB46//VAV3//TSLP//PIK3AP1//ARPC3//BAIAP2//AGO1//GABPA//POU4F1//SLAMF6//RHOA//RNF26//CD300LG//TNFAIP8L2//SIRPB1//SIRPG// |
| GO:0045672 | positive regulation of osteoclast differentiation | Biological process | 2 | 24 | 175 | 17653 | 8.40619047619048 | 0.0233759402892323 | 1 | 1.63123091075655 | 0.0114285714285714 | CSF1//POU4F1// |
| GO:0060444 | branching involved in mammary gland duct morphogenesis | Biological process | 2 | 24 | 175 | 17653 | 8.40619047619048 | 0.0233759402892323 | 1 | 1.63123091075655 | 0.0114285714285714 | VDR//CSF1// |
| GO:1900181 | negative regulation of protein localization to nucleus | Biological process | 2 | 24 | 175 | 17653 | 8.40619047619048 | 0.0233759402892323 | 1 | 1.63123091075655 | 0.0114285714285714 | CHP1//TRIM29// |
| GO:0051239 | regulation of multicellular organismal process | Biological process | 40 | 2969 | 175 | 17653 | 1.35903382572295 | 0.0235551349291253 | 1 | 1.62791440368188 | 0.228571428571429 | FFAR3//EFNA1//STMN2//RIT2//NF2//AGO1//CSF1//ZBTB46//NPVF//RNF26//MAVS//TSLP//SLAMF6//HLA-DPA1//FA2H//CR1//MAFG//VDR//GABPA//RHOA//ZC4H2//POU4F1//LRG1//STIM1//WWC3//SEMA3F//CLEC9A//THY1//CAMSAP2//PSMC6//VANGL1//BAIAP2//LRP8//P2RY2//ATP1B2//CTNNA3//ASPH//FXYD2//SYT2//ZNF609// |
| GO:0050771 | negative regulation of axonogenesis | Biological process | 3 | 62 | 175 | 17653 | 4.88101382488479 | 0.0236342803364545 | 1 | 1.62645761742136 | 0.0171428571428571 | SEMA3F//RHOA//THY1// |
| GO:0060284 | regulation of cell development | Biological process | 15 | 858 | 175 | 17653 | 1.76353646353646 | 0.0237473307633803 | 1 | 1.62438519866574 | 0.0857142857142857 | STMN2//RIT2//NF2//RHOA//ZC4H2//SEMA3F//POU4F1//EFNA1//THY1//CAMSAP2//BAIAP2//LRP8//SYT2//CSF1//ZNF609// |
| GO:0006814 | sodium ion transport | Biological process | 6 | 223 | 175 | 17653 | 2.7141063420884 | 0.0239159043789387 | 1 | 1.62131319165356 | 0.0342857142857143 | CHP1//SLC9B1//SLC24A3//SCN4A//ATP1B2//FXYD2// |
| GO:0006906 | vesicle fusion | Biological process | 5 | 166 | 175 | 17653 | 3.03838209982788 | 0.0249505968305696 | 1 | 1.60291906137835 | 0.0285714285714286 | TBC1D9B//VAV3//SYT2//VAMP2//DOC2A// |
| GO:1902533 | positive regulation of intracellular signal transduction | Biological process | 17 | 1021 | 175 | 17653 | 1.67959143696656 | 0.025196835909131 | 1 | 1.59865399229307 | 0.0971428571428571 | PEA15//MAS1//MAP3K9//PIK3AP1//TSLP//PIM2//RHOA//NDFIP2//MAVS//EFNA1//RIT2//MAPK8IP1//EEF1E1//CSF1//CCL16//AGO1//TRIM44// |
| GO:0002230 | positive regulation of defense response to virus by host | Biological process | 2 | 25 | 175 | 17653 | 8.06994285714286 | 0.0252455032356809 | 1 | 1.59781596779674 | 0.0114285714285714 | TRIM44//MAVS// |
| GO:0015012 | heparan sulfate proteoglycan biosynthetic process | Biological process | 2 | 25 | 175 | 17653 | 8.06994285714286 | 0.0252455032356809 | 1 | 1.59781596779674 | 0.0114285714285714 | HS3ST3B1//XYLT2// |
| GO:0046885 | regulation of hormone biosynthetic process | Biological process | 2 | 25 | 175 | 17653 | 8.06994285714286 | 0.0252455032356809 | 1 | 1.59781596779674 | 0.0114285714285714 | VDR//FFAR3// |
| GO:0002684 | positive regulation of immune system process | Biological process | 18 | 1105 | 175 | 17653 | 1.64320103425986 | 0.026109710885206 | 1 | 1.58319793712592 | 0.102857142857143 | MAVS//PSMC6//CR1//THY1//FFAR3//CSF1//HLA-DPA1//VAV3//TSLP//PIK3AP1//ARPC3//BAIAP2//POU4F1//SLAMF6//RHOA//SIRPB1//SIRPG//ZBTB46// |
| GO:0030004 | cellular monovalent inorganic cation homeostasis | Biological process | 4 | 114 | 175 | 17653 | 3.53944862155388 | 0.0268375640147622 | 1 | 1.57125690664606 | 0.0228571428571429 | ATP1B2//MAFG//CHP1//SLC9B1// |
| GO:0002360 | T cell lineage commitment | Biological process | 2 | 26 | 175 | 17653 | 7.75956043956044 | 0.0271738523837137 | 1 | 1.5658487881412 | 0.0114285714285714 | RHOA//SLAMF6// |
| GO:0022011 | myelination in peripheral nervous system | Biological process | 2 | 26 | 175 | 17653 | 7.75956043956044 | 0.0271738523837137 | 1 | 1.5658487881412 | 0.0114285714285714 | FA2H//CNTNAP1// |
| GO:0032292 | peripheral nervous system axon ensheathment | Biological process | 2 | 26 | 175 | 17653 | 7.75956043956044 | 0.0271738523837137 | 1 | 1.5658487881412 | 0.0114285714285714 | CNTNAP1//FA2H// |
| GO:0071407 | cellular response to organic cyclic compound | Biological process | 11 | 572 | 175 | 17653 | 1.93989010989011 | 0.0274971331305274 | 1 | 1.56071258364231 | 0.0628571428571429 | AGO1//NR6A1//RHOA//NCOA4//UFM1//ESRRG//VDR//MAVS//POU4F1//LRP8//GABPA// |
| GO:0050769 | positive regulation of neurogenesis | Biological process | 9 | 430 | 175 | 17653 | 2.11132225913621 | 0.0278367556690368 | 1 | 1.55538138246448 | 0.0514285714285714 | STMN2//RIT2//RHOA//ZC4H2//BAIAP2//LRP8//SYT2//CSF1//ZNF609// |
| GO:0090174 | organelle membrane fusion | Biological process | 5 | 171 | 175 | 17653 | 2.94954051796157 | 0.0278887698901599 | 1 | 1.55457064104759 | 0.0285714285714286 | VAV3//SYT2//VAMP2//DOC2A//TBC1D9B// |
| GO:0000122 | negative regulation of transcription by RNA polymerase II | Biological process | 14 | 800 | 175 | 17653 | 1.7653 | 0.0282409311162958 | 1 | 1.54912098849832 | 0.08 | EFNA1//CBX5//TRIM29//GABPA//SPDEF//NR6A1//PROX2//MYPOP//POU4F1//WWC3//GATAD2B//ZNF608//VDR//LRP8// |
| GO:0006810 | transport | Biological process | 62 | 5048 | 175 | 17653 | 1.23894724926421 | 0.028808082122136 | 1 | 1.54048565360776 | 0.354285714285714 | STIM1//VAMP2//MAPK8IP1//VAV3//SYT2//DOC2A//CHP1//ZDHHC19//ZDHHC20//NCOA4//SLC17A5//SLC24A3//SCN4A//VDR//NDFIP2//STARD3//SLC25A45//SLC43A2//KDELR1//TOM1L2//TBC1D9B//RAB9B//RAB2A//VIPAS39//CDK16//DNAJC5//ZW10//ARF3//SCAMP2//STEAP2//LRP8//CLEC9A//ANP32E//TMEM201//SLC37A4//PEA15//ATP1B2//FXYD2//FFAR3//ANKRD50//SLC9B1//ABCD3//PITPNM2//ARHGAP1//SMPD3//RIT2//GRPEL2//NPVF//MAVS//GRIA3//ASPH//ARPC3//BAIAP2//SIRPB1//LRG1//CR1//RHOA//SIRPG//THY1//PSMC6//P2RY2//TSLP// |
| GO:0031114 | regulation of microtubule depolymerization | Biological process | 2 | 27 | 175 | 17653 | 7.47216931216931 | 0.0291596527054335 | 1 | 1.53521765281675 | 0.0114285714285714 | STMN2//CAMSAP2// |
| GO:0009755 | hormone-mediated signaling pathway | Biological process | 6 | 234 | 175 | 17653 | 2.58652014652015 | 0.0293484538584043 | 1 | 1.53241477340957 | 0.0342857142857143 | NR6A1//RHOA//NCOA4//UFM1//ESRRG//VDR// |
| GO:0050688 | regulation of defense response to virus | Biological process | 3 | 68 | 175 | 17653 | 4.45033613445378 | 0.0300082507013308 | 1 | 1.52275932056655 | 0.0171428571428571 | TRIM44//MAVS//RNF26// |
| GO:0060998 | regulation of dendritic spine development | Biological process | 3 | 68 | 175 | 17653 | 4.45033613445378 | 0.0300082507013308 | 1 | 1.52275932056655 | 0.0171428571428571 | EFNA1//BAIAP2//LRP8// |
| GO:1901264 | carbohydrate derivative transport | Biological process | 3 | 69 | 175 | 17653 | 4.38583850931677 | 0.0311500462273036 | 1 | 1.50654130450247 | 0.0171428571428571 | SLC17A5//SLC37A4//P2RY2// |
| GO:0048489 | synaptic vesicle transport | Biological process | 4 | 120 | 175 | 17653 | 3.36247619047619 | 0.0315833511195935 | 1 | 1.50054179150523 | 0.0228571428571429 | SYT2//VAMP2//DNAJC5//DOC2A// |
| GO:0097480 | establishment of synaptic vesicle localization | Biological process | 4 | 120 | 175 | 17653 | 3.36247619047619 | 0.0315833511195935 | 1 | 1.50054179150523 | 0.0228571428571429 | SYT2//VAMP2//DNAJC5//DOC2A// |
| GO:0050808 | synapse organization | Biological process | 7 | 304 | 175 | 17653 | 2.32276315789474 | 0.0321406238070709 | 1 | 1.49294569840841 | 0.04 | POU4F1//PDZRN3//ZC4H2//EFNA1//BAIAP2//LRP8//RHOA// |
| GO:0097061 | dendritic spine organization | Biological process | 3 | 70 | 175 | 17653 | 4.32318367346939 | 0.032314423200925 | 1 | 1.49060359164882 | 0.0171428571428571 | EFNA1//BAIAP2//LRP8// |
| GO:0043687 | post-translational protein modification | Biological process | 9 | 442 | 175 | 17653 | 2.05400129282482 | 0.0323753747497504 | 1 | 1.48978519585875 | 0.0514285714285714 | TECTB//THY1//KLHL2//CSF1//IGFBP7//PSMC6//RAB2A//VIPAS39//DDA1// |
| GO:0014044 | Schwann cell development | Biological process | 2 | 29 | 175 | 17653 | 6.95684729064039 | 0.0332983686139806 | 1 | 1.47757704334958 | 0.0114285714285714 | CNTNAP1//FA2H// |
| GO:0070306 | lens fiber cell differentiation | Biological process | 2 | 29 | 175 | 17653 | 6.95684729064039 | 0.0332983686139806 | 1 | 1.47757704334958 | 0.0114285714285714 | PROX2//NF2// |
| GO:0046785 | microtubule polymerization | Biological process | 3 | 71 | 175 | 17653 | 4.26229376257545 | 0.0335013214115465 | 1 | 1.47493806250271 | 0.0171428571428571 | CAMSAP2//STMN2//TPPP3// |
| GO:0098739 | import across plasma membrane | Biological process | 3 | 71 | 175 | 17653 | 4.26229376257545 | 0.0335013214115465 | 1 | 1.47493806250271 | 0.0171428571428571 | STEAP2//ARHGAP1//FXYD2// |
| GO:0042476 | odontogenesis | Biological process | 4 | 123 | 175 | 17653 | 3.28046457607433 | 0.0341296265101263 | 1 | 1.46686846429911 | 0.0228571428571429 | NF2//CSF1//STIM1//RHOA// |
| GO:0031328 | positive regulation of cellular biosynthetic process | Biological process | 26 | 1812 | 175 | 17653 | 1.44742352570167 | 0.0345082755174122 | 1 | 1.46207674317113 | 0.148571428571429 | RHOA//PIM2//ESRRG//TRIM44//ANKRD49//NCOA4//MAS1//PFKFB3//PSMC6//PRDM4//ZNF609//GABPA//SPDEF//NR6A1//AGO1//PROX2//HLF//MAFG//POU4F1//MAVS//RIT2//TEAD3//VDR//ZNF24//CHP1//SMPD3// |
| GO:1904064 | positive regulation of cation transmembrane transport | Biological process | 4 | 124 | 175 | 17653 | 3.25400921658986 | 0.0350042144242605 | 1 | 1.45587966447789 | 0.0228571428571429 | STIM1//CHP1//THY1//ATP1B2// |
| GO:0106027 | neuron projection organization | Biological process | 3 | 73 | 175 | 17653 | 4.14551859099804 | 0.0359424116625935 | 1 | 1.44439278597447 | 0.0171428571428571 | EFNA1//BAIAP2//LRP8// |
| GO:0038061 | NIK/NF-kappaB signaling | Biological process | 4 | 126 | 175 | 17653 | 3.20235827664399 | 0.0367922245010751 | 1 | 1.43424395342096 | 0.0228571428571429 | MAS1//AGO1//TRIM44//PSMC6// |
| GO:0045664 | regulation of neuron differentiation | Biological process | 11 | 600 | 175 | 17653 | 1.8493619047619 | 0.0369732349190277 | 1 | 1.43211254980501 | 0.0628571428571429 | STMN2//RIT2//RHOA//ZC4H2//SEMA3F//EFNA1//THY1//CAMSAP2//BAIAP2//LRP8//SYT2// |
| GO:0032642 | regulation of chemokine production | Biological process | 3 | 74 | 175 | 17653 | 4.08949806949807 | 0.0371964547394411 | 1 | 1.4294984515286 | 0.0171428571428571 | FFAR3//TSLP//MAVS// |
| GO:0010765 | positive regulation of sodium ion transport | Biological process | 2 | 31 | 175 | 17653 | 6.50801843317972 | 0.0376513744045811 | 1 | 1.42421916593258 | 0.0114285714285714 | CHP1//ATP1B2// |
| GO:0030201 | heparan sulfate proteoglycan metabolic process | Biological process | 2 | 31 | 175 | 17653 | 6.50801843317972 | 0.0376513744045811 | 1 | 1.42421916593258 | 0.0114285714285714 | XYLT2//HS3ST3B1// |
| GO:0097479 | synaptic vesicle localization | Biological process | 4 | 127 | 175 | 17653 | 3.17714285714286 | 0.0377056692828189 | 1 | 1.42359334599749 | 0.0228571428571429 | SYT2//VAMP2//DNAJC5//DOC2A// |
| GO:0021766 | hippocampus development | Biological process | 3 | 75 | 175 | 17653 | 4.03497142857143 | 0.0384727214497701 | 1 | 1.41484709186281 | 0.0171428571428571 | LRP8//MAS1//NF2// |
| GO:0098876 | vesicle-mediated transport to the plasma membrane | Biological process | 3 | 75 | 175 | 17653 | 4.03497142857143 | 0.0384727214497701 | 1 | 1.41484709186281 | 0.0171428571428571 | STEAP2//VAMP2//ANKRD50// |
| GO:0006367 | transcription initiation from RNA polymerase II promoter | Biological process | 5 | 187 | 175 | 17653 | 2.69717341482047 | 0.0387290840119687 | 1 | 1.41196277446374 | 0.0285714285714286 | PSMC6//ESRRG//NR6A1//TEAD3//VDR// |
| GO:0034764 | positive regulation of transmembrane transport | Biological process | 5 | 187 | 175 | 17653 | 2.69717341482047 | 0.0387290840119687 | 1 | 1.41196277446374 | 0.0285714285714286 | STIM1//CHP1//THY1//ATP1B2//FFAR3// |
| GO:0007031 | peroxisome organization | Biological process | 2 | 32 | 175 | 17653 | 6.30464285714286 | 0.0399051106763973 | 1 | 1.39897148034275 | 0.0114285714285714 | MAVS//ABCD3// |
| GO:0014072 | response to isoquinoline alkaloid | Biological process | 2 | 32 | 175 | 17653 | 6.30464285714286 | 0.0399051106763973 | 1 | 1.39897148034275 | 0.0114285714285714 | MAP1LC3A//PEA15// |
| GO:0043278 | response to morphine | Biological process | 2 | 32 | 175 | 17653 | 6.30464285714286 | 0.0399051106763973 | 1 | 1.39897148034275 | 0.0114285714285714 | MAP1LC3A//PEA15// |
| GO:0060603 | mammary gland duct morphogenesis | Biological process | 2 | 32 | 175 | 17653 | 6.30464285714286 | 0.0399051106763973 | 1 | 1.39897148034275 | 0.0114285714285714 | CSF1//VDR// |
| GO:0032273 | positive regulation of protein polymerization | Biological process | 4 | 130 | 175 | 17653 | 3.10382417582418 | 0.0405238546197415 | 1 | 1.39228925135559 | 0.0228571428571429 | BAIAP2//RHOA//ARPC3//TPPP3// |
| GO:0061025 | membrane fusion | Biological process | 6 | 253 | 175 | 17653 | 2.39227555053642 | 0.0405298823472433 | 1 | 1.39222465695451 | 0.0342857142857143 | VAV3//SYT2//VAMP2//DOC2A//TBC1D9B//CHP1// |
| GO:0009891 | positive regulation of biosynthetic process | Biological process | 26 | 1841 | 175 | 17653 | 1.42462326375417 | 0.0407203241143651 | 1 | 1.39018877388053 | 0.148571428571429 | RHOA//PIM2//ESRRG//TRIM44//ANKRD49//NCOA4//MAS1//PFKFB3//PSMC6//PRDM4//ZNF609//GABPA//SPDEF//NR6A1//AGO1//PROX2//HLF//MAFG//POU4F1//MAVS//RIT2//TEAD3//VDR//ZNF24//CHP1//SMPD3// |
| GO:0007422 | peripheral nervous system development | Biological process | 3 | 77 | 175 | 17653 | 3.93016697588126 | 0.0410915696038043 | 1 | 1.38624726936719 | 0.0171428571428571 | CNTNAP1//FA2H//POU4F1// |
| GO:0061097 | regulation of protein tyrosine kinase activity | Biological process | 3 | 77 | 175 | 17653 | 3.93016697588126 | 0.0410915696038043 | 1 | 1.38624726936719 | 0.0171428571428571 | EFNA1//LRP8//THY1// |
| GO:0050863 | regulation of T cell activation | Biological process | 7 | 321 | 175 | 17653 | 2.1997507788162 | 0.041177511573487 | 1 | 1.38533990207343 | 0.04 | HLA-DPA1//CR1//RHOA//TNFAIP8L2//SIRPB1//SIRPG//THY1// |
| GO:0009967 | positive regulation of signal transduction | Biological process | 23 | 1586 | 175 | 17653 | 1.46286795172041 | 0.0413158999031601 | 1 | 1.38388278342017 | 0.131428571428571 | PEA15//TRIM44//TSLP//MAS1//MAP3K9//PIK3AP1//LRG1//PIM2//RHOA//NDFIP2//MAVS//EFNA1//RIT2//MAPK8IP1//EEF1E1//CSF1//CCL16//PSMC6//AGO1//BAIAP2//VAV3//ARHGAP1//CNTNAP1// |
| GO:0042110 | T cell activation | Biological process | 9 | 463 | 175 | 17653 | 1.96083924714594 | 0.0415134032099502 | 1 | 1.38181166231605 | 0.0514285714285714 | RHOA//KDELR1//HLA-DPA1//CR1//TNFAIP8L2//SIRPB1//SIRPG//THY1//SLAMF6// |
| GO:0032409 | regulation of transporter activity | Biological process | 6 | 255 | 175 | 17653 | 2.37351260504202 | 0.0418440901925122 | 1 | 1.37836587047879 | 0.0342857142857143 | STIM1//NDFIP2//CHP1//ATP1B2//VAMP2//FXYD2// |
| GO:0018345 | protein palmitoylation | Biological process | 2 | 33 | 175 | 17653 | 6.11359307359307 | 0.0422087075486423 | 1 | 1.37459794595235 | 0.0114285714285714 | ZDHHC19//ZDHHC20// |
| GO:0030224 | monocyte differentiation | Biological process | 2 | 33 | 175 | 17653 | 6.11359307359307 | 0.0422087075486423 | 1 | 1.37459794595235 | 0.0114285714285714 | ZBTB46//CSF1// |
| GO:0031111 | negative regulation of microtubule polymerization or depolymerization | Biological process | 2 | 33 | 175 | 17653 | 6.11359307359307 | 0.0422087075486423 | 1 | 1.37459794595235 | 0.0114285714285714 | STMN2//CAMSAP2// |
| GO:0032350 | regulation of hormone metabolic process | Biological process | 2 | 33 | 175 | 17653 | 6.11359307359307 | 0.0422087075486423 | 1 | 1.37459794595235 | 0.0114285714285714 | FFAR3//VDR// |
| GO:1903131 | mononuclear cell differentiation | Biological process | 2 | 33 | 175 | 17653 | 6.11359307359307 | 0.0422087075486423 | 1 | 1.37459794595235 | 0.0114285714285714 | ZBTB46//CSF1// |
| GO:0051234 | establishment of localization | Biological process | 62 | 5155 | 175 | 17653 | 1.21323098240266 | 0.0429864245296767 | 1 | 1.36666867657661 | 0.354285714285714 | ZW10//STIM1//VAMP2//MAPK8IP1//VAV3//SYT2//DOC2A//CHP1//ZDHHC19//ZDHHC20//NCOA4//SLC17A5//SLC24A3//SCN4A//VDR//NDFIP2//STARD3//SLC25A45//SLC43A2//KDELR1//TOM1L2//TBC1D9B//RAB9B//RAB2A//VIPAS39//CDK16//DNAJC5//ARF3//SCAMP2//STEAP2//LRP8//CLEC9A//ANP32E//TMEM201//SLC37A4//PEA15//ATP1B2//FXYD2//FFAR3//ANKRD50//SLC9B1//ABCD3//PITPNM2//ARHGAP1//SMPD3//RIT2//GRPEL2//NPVF//MAVS//GRIA3//ASPH//ARPC3//BAIAP2//SIRPB1//LRG1//CR1//RHOA//SIRPG//THY1//PSMC6//P2RY2//TSLP// |
| GO:0099504 | synaptic vesicle cycle | Biological process | 4 | 133 | 175 | 17653 | 3.03381310418904 | 0.0434588808830009 | 1 | 1.36192146146223 | 0.0228571428571429 | SYT2//VAMP2//DNAJC5//DOC2A// |
| GO:1902107 | positive regulation of leukocyte differentiation | Biological process | 4 | 133 | 175 | 17653 | 3.03381310418904 | 0.0434588808830009 | 1 | 1.36192146146223 | 0.0228571428571429 | CSF1//POU4F1//RHOA//ZBTB46// |
| GO:1901879 | regulation of protein depolymerization | Biological process | 3 | 79 | 175 | 17653 | 3.83066907775769 | 0.0437981943019118 | 1 | 1.35854379408222 | 0.0171428571428571 | STMN2//CAMSAP2//ASPH// |
| GO:0050768 | negative regulation of neurogenesis | Biological process | 6 | 259 | 175 | 17653 | 2.33685603971318 | 0.0445525966911352 | 1 | 1.35112697859082 | 0.0342857142857143 | STMN2//RIT2//SEMA3F//RHOA//THY1//EFNA1// |
| GO:0050775 | positive regulation of dendrite morphogenesis | Biological process | 2 | 34 | 175 | 17653 | 5.93378151260504 | 0.0445609674145474 | 1 | 1.3510453892383 | 0.0114285714285714 | BAIAP2//LRP8// |
| GO:1902905 | positive regulation of supramolecular fiber organization | Biological process | 5 | 195 | 175 | 17653 | 2.58652014652015 | 0.0449931342927399 | 1 | 1.34685375214164 | 0.0285714285714286 | BAIAP2//RHOA//STMN2//ARPC3//NF2// |
| GO:0010976 | positive regulation of neuron projection development | Biological process | 6 | 260 | 175 | 17653 | 2.32786813186813 | 0.0452464883749507 | 1 | 1.34441512117837 | 0.0342857142857143 | RHOA//BAIAP2//LRP8//SYT2//STMN2//RIT2// |
| GO:0042063 | gliogenesis | Biological process | 6 | 260 | 175 | 17653 | 2.32786813186813 | 0.0452464883749507 | 1 | 1.34441512117837 | 0.0342857142857143 | HDAC11//NF2//RHOA//CNTNAP1//FA2H//CSF1// |
| GO:0071383 | cellular response to steroid hormone stimulus | Biological process | 6 | 260 | 175 | 17653 | 2.32786813186813 | 0.0452464883749507 | 1 | 1.34441512117837 | 0.0342857142857143 | NR6A1//RHOA//NCOA4//UFM1//ESRRG//VDR// |
| GO:0032870 | cellular response to hormone stimulus | Biological process | 12 | 698 | 175 | 17653 | 1.73422840769546 | 0.0454379311072603 | 1 | 1.34258145126613 | 0.0685714285714286 | BAIAP2//NR6A1//RHOA//NCOA4//VAMP2//UFM1//MAS1//ESRRG//VDR//POU4F1//FFAR3//IGFBP7// |
| GO:0048284 | organelle fusion | Biological process | 5 | 196 | 175 | 17653 | 2.57332361516035 | 0.0458163280251027 | 1 | 1.33897972052285 | 0.0285714285714286 | VAV3//SYT2//VAMP2//DOC2A//TBC1D9B// |
| GO:0014070 | response to organic cyclic compound | Biological process | 15 | 937 | 175 | 17653 | 1.61484982466839 | 0.0459596825475352 | 1 | 1.33762297972983 | 0.0857142857142857 | AGO1//NR6A1//RHOA//NCOA4//UFM1//MAP1LC3A//PEA15//ESRRG//VDR//IGFBP7//MAVS//POU4F1//LRP8//GABPA//ABCD3// |
| GO:0034767 | positive regulation of ion transmembrane transport | Biological process | 4 | 136 | 175 | 17653 | 2.96689075630252 | 0.0465106959922158 | 1 | 1.3324471615997 | 0.0228571428571429 | STIM1//CHP1//THY1//ATP1B2// |
| GO:0032602 | chemokine production | Biological process | 3 | 81 | 175 | 17653 | 3.73608465608466 | 0.0465917578650579 | 1 | 1.33169090370304 | 0.0171428571428571 | FFAR3//TSLP//MAVS// |
| GO:0065008 | regulation of biological quality | Biological process | 47 | 3770 | 175 | 17653 | 1.25758393330807 | 0.0480893620467547 | 1 | 1.31795098423648 | 0.268571428571429 | RHOA//CBX5//MAFG//TXNDC8//TMX1//KDELR1//STARD3//P2RY2//SLC24A3//STIM1//VDR//NCOA4//ATP1B2//VAMP2//DNAJC5//BAIAP2//FGD1//VAV3//SYT2//PIM2//CHP1//EFNA1//TRIM44//CNTNAP1//SCN4A//SMPD3//GNA14//CDK16//ASPH//NF2//NPVF//RIT2//ARPC3//SAMD4B//PSMC6//FFAR3//DEPTOR//DOC2A//SEMA3F//CSF1//THY1//SLC9B1//STEAP2//GRIA3//LRP8//CTNNA3//IARS// |
| GO:0010557 | positive regulation of macromolecule biosynthetic process | Biological process | 24 | 1698 | 175 | 17653 | 1.42578495709238 | 0.0481157512240078 | 1 | 1.31771272924232 | 0.137142857142857 | RHOA//PIM2//ESRRG//TRIM44//ANKRD49//NCOA4//MAS1//PSMC6//PRDM4//ZNF609//GABPA//SPDEF//NR6A1//AGO1//PROX2//HLF//MAFG//POU4F1//MAVS//RIT2//TEAD3//VDR//ZNF24//CHP1// |
| GO:0030518 | intracellular steroid hormone receptor signaling pathway | Biological process | 4 | 138 | 175 | 17653 | 2.92389233954451 | 0.0486100247948658 | 1 | 1.31327415740168 | 0.0228571428571429 | RHOA//NCOA4//UFM1//NR6A1// |
| GO:0050919 | negative chemotaxis | Biological process | 2 | 36 | 175 | 17653 | 5.60412698412698 | 0.0494067784698067 | 1 | 1.30621346301806 | 0.0114285714285714 | RHOA//SEMA3F// |
| GO:0006890 | retrograde vesicle-mediated transport, Golgi to ER | Biological process | 3 | 83 | 175 | 17653 | 3.64605851979346 | 0.0494713513732809 | 1 | 1.30564622616416 | 0.0171428571428571 | KDELR1//ARF3//ZW10// |
| GO:0042446 | hormone biosynthetic process | Biological process | 3 | 83 | 175 | 17653 | 3.64605851979346 | 0.0494713513732809 | 1 | 1.30564622616416 | 0.0171428571428571 | STARD3//FFAR3//VDR// |
| GO:0048814 | regulation of dendrite morphogenesis | Biological process | 3 | 83 | 175 | 17653 | 3.64605851979346 | 0.0494713513732809 | 1 | 1.30564622616416 | 0.0171428571428571 | EFNA1//BAIAP2//LRP8// |
| GO:0099003 | vesicle-mediated transport in synapse | Biological process | 4 | 139 | 175 | 17653 | 2.90285714285714 | 0.0496790900718589 | 1 | 1.30382636734818 | 0.0228571428571429 | SYT2//VAMP2//DNAJC5//DOC2A// |
